# Tumor Stiffness Is Unrelated to Myosin Light Chain Phosphorylation in Cancer Cells

**DOI:** 10.1371/journal.pone.0079776

**Published:** 2013-11-04

**Authors:** Hui-Jun Yu, Leonid A. Serebryannyy, Madeline Fry, Madelyne Greene, Olga Chernaya, Wen-Yang Hu, Teng-Leong Chew, Nadim Mahmud, Shrihari S. Kadkol, Sarah Glover, Gail Prins, Zuzana Strakova, Primal de Lanerolle

**Affiliations:** 1 Department of Physiology and Biophysics, University of Illinois at Chicago, Chicago, Illinois, United States of America; 2 Department of Urology, University of Illinois at Chicago, Chicago, Illinois, United States of America; 3 Department of Cell Biology, Northwestern University, Chicago, Illinois, United States of America; 4 Department of Hematology and Oncology, University of Illinois at Chicago, Chicago, Illinois, United States of America; 5 Department of Pathology, University of Illinois at Chicago, Chicago, Illinois, United States of America; 6 Department of Medicine, University of Illinois at Chicago, Chicago, Illinois, United States of America; 7 Department of Obstetrics and Gynecology, University of Illinois at Chicago, Chicago, Illinois, United States of America; Beatson Institute for Cancer Research Glasgow, United Kingdom

## Abstract

Many tumors are stiffer than their surrounding tissue. This increase in stiffness has been attributed, in part, to a Rho-dependent elevation of myosin II light chain phosphorylation. To characterize this mechanism further, we studied myosin light chain kinase (MLCK), the main enzyme that phosphorylates myosin II light chains. We anticipated that increases in MLCK expression and activity would contribute to the increased stiffness of cancer cells. However, we find that MLCK mRNA and protein levels are substantially less in cancer cells and tissues than in normal cells. Consistent with this observation, cancer cells contract 3D collagen matrices much more slowly than normal cells. Interestingly, inhibiting MLCK or Rho kinase did not affect the 3D gel contractions while blebbistatin partially and cytochalasin D maximally inhibited contractions. Live cell imaging of cells in collagen gels showed that cytochalasin D inhibited filopodia-like projections that formed between cells while a MLCK inhibitor had no effect on these projections. These data suggest that myosin II phosphorylation is dispensable in regulating the mechanical properties of tumors.

## Introduction

Many types of tumors can be detected by palpation because they are stiffer or harder than the surrounding tissue. The mechanical properties of a tumor are determined by the combined effects and interactions of multiple parameters [1]. The stroma, the composition and stiffness of the extracellular matrix, integrin ligation, increased vascularization, fluid accumulation and the presence of immune cells such as macrophages contribute to the overall stiffness of the tumor [1-3]. The physical characteristics of the transformed cells, which can be affected by the genetic signature of the tumor cells [4] and the microenvironment [5,6] also play a part in determining tumor stiffness. Cell stiffness is primarily determined by actin-myosin II interactions [7,8]. The actin-myosin II interaction in non-muscle cells is regulated by the phosphorylation of myosin light chains (MLC) [9]. Actin and phospho-myosin II comprise the molecular motor that converts ATP into mechanical work in smooth muscle and non-muscle cells [9-11] and an increase in MLC phosphorylation has been implicated in determining tumor stiffness [1,2].

There are two major pathways that regulate MLC phosphorylation. One pathway involves myosin light chain kinase (MLCK). MLCK is a calcium-calmodulin dependent enzyme that phosphorylates the regulatory light chain of smooth muscle and non-muscle myosin II [9,10]. Unlike other protein kinases that phosphorylate multiple substrates, MLC appear to be the sole substrate for MLCK. MLC phosphorylation/dephosphorylation regulates smooth muscle contraction [9] and many other energy-dependent processes, including cell division [10] and cell motility [11,12]. Because cell proliferation and metastatic colonization are two of the most pernicious aspects of cancer, it is reasonable to predict an important role for MLCK in tumor growth and metastatic colonization. In support of this idea, MLCK has been implicated in cell survival [13,14] and inhibiting MLCK has been shown to induce apoptosis [13,15] and to decrease tumor growth [15]. Decreased MLC phosphorylation has also been implicated in cytokinesis failure in cancer cells [16].

The second pathway involves the Rho A GTPase mediated the activation of Rho kinase or ROCK. While the phosphorylation of MLC by ROCK has been reported, ROCK appears to increase MLC phosphorylation mainly by phosphorylating and inactivating a myosin phosphatase [17]. Because the level of MLC phosphorylation represents a balance between the enzymes that phosphorylate and dephosphorylate MLC, inhibiting myosin phosphatase increases the intracellular level of MLC phosphorylation [17]. The Rho/ROCK pathway plays a crucial role in communicating extracellular signals that affect the nature of the cytoskeleton, especially signals from the extracellular matrix that result in increased cell tension [18]. This pathway is also central in regulating cell motility and cancer metastasis [12]. Blocking ROCK has been shown to inhibit tumor growth and progression [2] and, even though Rho A is not an oncogene, an increase in Rho A expression is detected in cancer and the Rho A/ROCK pathway is implicated in Ras-mediated transformation [4].

Thus, there is a wealth of data demonstrating that MLC phosphorylation is a focal point in the transformation process, the response of cancer cells to the extracellular matrix and the proliferation and migration of cancer cells. To understand the importance of the two major signaling pathways that regulate MLC phosphorylation, we investigated the expression of MLCK in cancer cells. Our hypothesis was an increase in MLCK expression in cancer cells would result in increased cytoskeletal tension and cellular contractile responses. To our surprise, we have found that cancer tissues and cells express less MLCK than their normal counterparts and normal cells contract 3D collagen gels more rapidly than cancer cells. Furthermore, blocking MLCK or ROCK has no effect on 3D gel contractions whereas cytochalasin D, which disrupts actin filaments, blocked these contractions.

## Methods

### Cells and Tissue Culturing

Mononuclear cells (MNC) (<1.077 g/ml) were obtained by density centrifugation on Ficoll-Paque PLUS (GE Healthcare Bio-Sciences AB, Uppsala, Sweden) as described previously [19]. Human uterine fibroblasts (HUF cells) were isolated as previously described [20]. The following human cells, obtained commercially (source and catalog number included), were also used: HeLa cervical cancer cells (ATCC, CCL-2), ECC-1 endometrial epithelial adenocarcinoma cells (ATCC, CRL-2923), primary prostate epithelial cells (1° prostate) from disease-free men, LNCaP prostate cancer cells (Lonza, CC-25555), HCT116 colon cancer cells (ATCC, CCL-247), MCF10A non-transformed mammary epithelial cells (ATCC, CRL-10317), MCF-7 (ATCC, HTB-22) and T47D (ATCC, CRL-2865) mammary cancer cells, H520 squamous lung cancer cells (ATCC, HTB-182), Hec-1A endometrial adenocarcinoma cells (ATCC, HTB-113), HEK293T immortalized kidney cells (ATCC, CRL-11268), Beas-2B transformed lung bronchial epithelial cells (ATCC, CRL-9609), human umbilical vein endothelial cells (HUVEC) (Lonza, CC-2517), human pulmonary artery endothelial cells (HPAEC) (Lonza, CC-2530), human normal pulmonary artery smooth muscle cells (HPASMC) (Lonza, CC-2581) and human normal lung microvasculature endothelial cells (HLMEC) (Lonza, CC-2527). We used MCF10A and Beas-2B cells as controls because, while they grow continuously in culture, they do not form tumors when injected into immunodeficient mice [21,22]. Five pairs of human cancer tissues (bladder, colon, lung, ovary, and uterus) and surrounding normal tissue were obtained from the Cooperative Human Tissue Network, Midwest Division (Columbus, OH) and stored frozen at liquid nitrogen. Each pair of tissues was from the same patient.

### RNA Isolation and PCR

Total RNA was isolated from cells and tissues using Trizol as recommended by the manufacturer (Invitrogen, Carlsbad, CA). RNA was reverse transcribed with SuperScript III reverse transcriptase (Invitrogen, Carlsbad, CA) and RT-PCR was performed using 2 μl of cDNA and 0.5 μM, each, of the 3bf forward primer and the 3ar backward primer described by Brand-Arpon et al. [23]. Quantitative PCR was performed using SYBR Green PCR Master Mix (Applied Biosystems, Foster City, CA) according to manufacturer’s directions. The primers for total MLCK P4610 (5’ AGG AGC CCG AGG TTG ATT AC 3’) and R4762 (5’ ACT TCC CTG CCC AGA CTT TT 3’) target exons 26 and 27. The specificity of primers was validated by a dissociation curve analysis and the fold change in expression of each gene was calculated using the ΔΔCt method, with H3F3A as an internal control.

### Western Blot Analyses

Pieces of each of the frozen tumor and normal tissue were excised while frozen, homogenized in 10X w/v hot SDS sample buffer and heated in a boiling water bath for 5 min. The supernatants were collected by centrifugation and 10 μl of each sample were applied to 4-20% polyacrylamide gradient SDS gels and transferred to nitrocellulose. The top half of each blot was probed with a rabbit, affinity purified antibody to MLCK [24] and the bottom was probed with an antibody to GAPDH. Cells were harvested, suspended in PBS, incubated with 2 mM diisopropylfluorophosphate for 10 mins at room temperature and extracted in 9M urea, 50 mM DTT, 50 mM Tris, pH 6.8. The supernatants were collected by centrifugation, protein concentrations were determined using a Bradford Assay and 10 μg of protein per sample were applied to 4-20% polyacrylamide gradient SDS gels and transferred to nitrocellulose.

### Measurement of MLC Phosphorylation

MLC phosphorylation was quantified in HUF and HeLa cells using urea/glycerol gels as described by Chew et al. [25] with slight modifications. Cells were treated with inhibitors for 2 hours, washed in isotonic sucrose 2X and extracted in 9M urea, 5 mM DTT, 20 mM Tris, pH 6.8. The supernatants were collected by centrifugation, protein concentrations were determined and 100 μg of HUF cell extract and 225 μg of HeLa cell extract were separated using glycerol-urea PAGE. The proteins were transferred to nitrocellulose, the un- and phosphorylated forms of MLC were identified using an antibody to MLC and the stoichiometry of phosphorylation (mol PO_4_/mol MLC_20_) was calculated as previously described [26].

### Collagen Gel Contraction Assay and Drug Treatment

To prepare the collagen gel solution, cell growth medium was supplemented with 1 mg/ml collagen Type 1 rat tail collage (BD Biosciences), neutralized with 1 N NaOH to pH 7.5 and buffered with 10 mM HEPES, was combined with tissue culture media and kept on ice. Cells were collected, counted and 5X10^5^ cells were resuspended in 200 μl of collagen solution and applied to individual wells of a 48-well plate (Fisher Scientific, Pittsburg, PA). The gels were incubated at 37 °C in a CO_2_ incubator for 15-20 min until they solidified and then detached from the walls of the wells with pipette tips. Growth medium (200 μl) was added to each well and the gels were allowed to contact for 18 hours or as specified. All the gels were photographed before and after contraction. The areas of the gels were measured in Image J and the percentage of the gel size after contraction, normalized to the cross-sectional area of the well, was calculated.

### Drug Treatments of Collagen Cells

ML-7, Y 27632 and blebbistatin were purchased from EMD Biosciences and cytochalasin D was purchased from Enzo Life Sciences. Drugs were diluted in growth medium and added to the gels. Drug concentrations were calculated based on the total volume of media and collagen gel. Untreated cells and cells treated with DMSO were used as controls.

### Live-Cell Imaging of 3D Collagen Gels

HeLa cells were transiently transfected by electroporation using a BioRad Gene Pulser Xcell with either with pLL7.0 mCherry-LifeAct (gift of Dr. Robert Wysolmasky) or pEGFP-LifeAct (gift from Alexander Bershadsky), which binds to F-actin [27]. Briefly, a 10 cm dish of 95% confluent Hela cells was trypsinized and resuspended in 10 ml growth media. Cells were spun down at 2000 Xg for 5 minutes and resuspended in 400 ul Opti-MEM® I Reduced Serum Medium supplemented with 4 ul of 1 M Hepes, 1ug of either LifeAct plasmid and 10ug of Sheared Salmon Sperm DNA. Electroporation was done in a 4 mm cuvette (BioRad using the following setting (Voltage=240 V, capacitance=950uF, resistance=∞). A 1:1 ratio of mCherry-LifeAct and EGFP-LifeAct transfected HeLa cells were mixed together and a total of 2.5 x 106 cells were mixed with 1 ml 1 mg/ml collagen [28]. Collagen-cells solution was added to the well of a glass bottom Mat Tek imaging dish (P35G-1.5-14-C), solidified for 20 minutes at room temperature and 2 ml growth media was added to cover the cell-gel matrix and placed in the 5% CO2 incubator. Appropriate inhibitors was added to the media and cells were imaged on Nikon A1 laser scanning confocal microscope, using Apo 100x1.45 N.A. objective, equipped with Tokai environmental chamber.

### Ethics Statement

Tissues were obtained from the Cooperative Human Tissue Network, Midwest Division (The Ohio State University, Columbus, OH), a NCI funded tissue repository (https://htrn.osu.edu). Other investigators may have received specimens from the same subjects. Human umbilical cord blood was obtained from the New York Blood Center (New York, NY, http:/nybloodcenter.org) according to Institutional Review Board (IRB) guidelines. Protocols for isolation of human CB CD34+ cells by NM were approved by the IRB of the University of Illinois at Chicago. HUF cells were isolated from the decidua peritalis dissected from placental membranes after normal vaginal delivery at term by ZS with prior approval from the IRB at the University of Illinois at Chicago. No animals were used.

## Results

The MYLK gene is located on chromosome 3q21 (GenBank Accession Number U48959) in humans. The MYLK gene spans >272 kb, contains at least 34 exons and codes for 3 proteins [29]: nmMLCK (~210 kD), smMLCK (~150 kD) and a small protein called telokin. Human nmMLCK and smMLCK are transcribed by exons 1-34 [23] and 18-34 [30], respectively. Analysis of the exon-intron structure in the human MYLK gene has revealed splice variants of nmMLCK that have unique localization patterns in epithelial cells [30]. At least four non-muscle MLCK isoforms (MLCK2, 3a, 3b and 4) that are the result of alternatively spliced variants of a mRNA precursor have been described [31]. In contrast, smMLCK, encoded by exons 18-34 [29], is expressed mainly in smooth muscle cells and low levels of smMLCK are detected in epithelial and endothelial cells (see below). Although smMLCK and nmMLCK are structurally different, both apparently only phosphorylate the regulatory light chain of smooth muscle and non-muscle myosin II [9].

**Figure 1:**
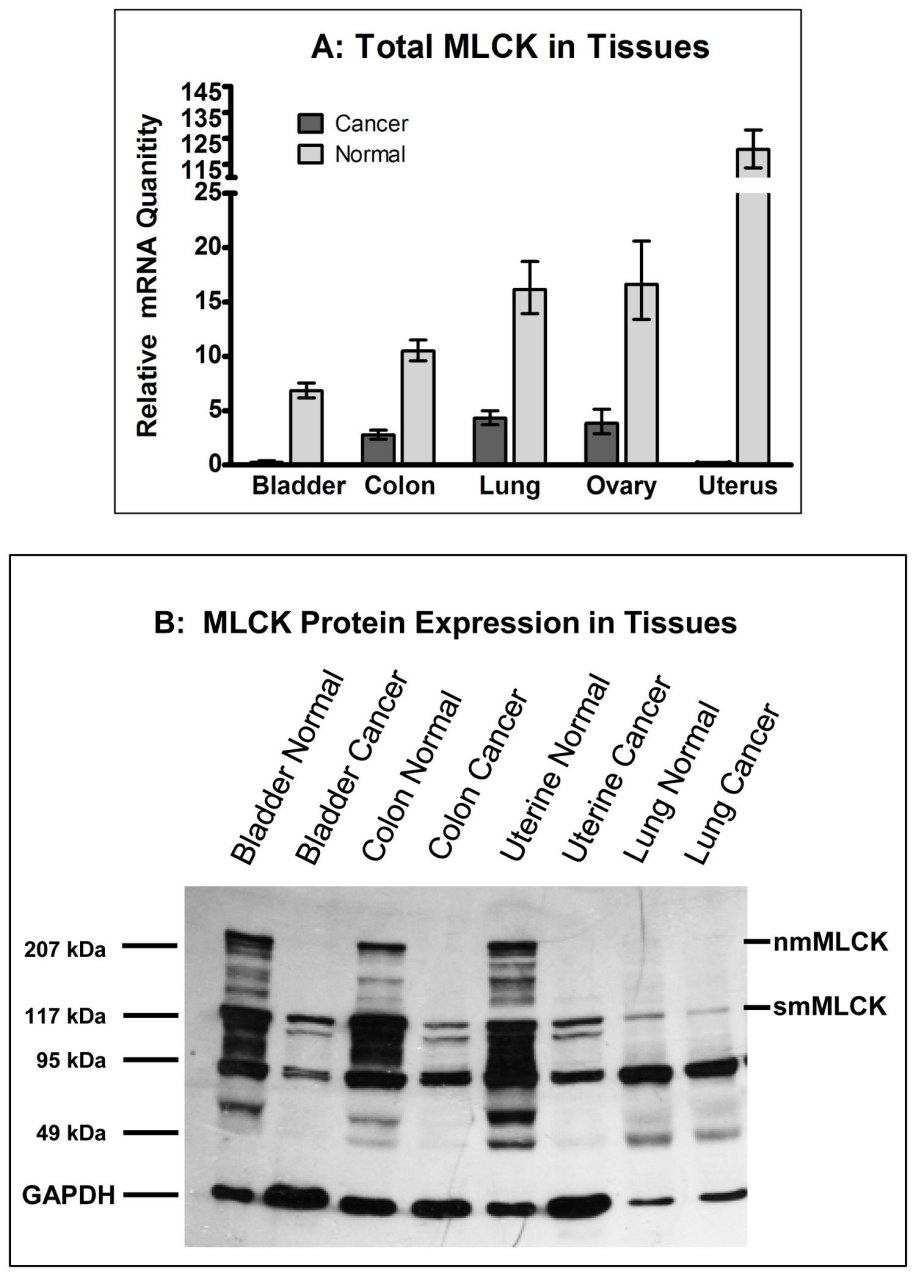
Analysis of MLCK expression in normal and cancer tissues. Quantitative PCR (A) and western blot (B) analyses of normal and cancer tissues. RNA was isolated from various normal and cancer tissues and total MLCK (A), including all splice variants of nmMLCK and smMLCK, was detected using primers targeting exons 26 and 27. The gene for H3F3A was used as an internal control in all experiments. The data in Panel A depict the averages of qPCR analyses performed in triplicate and the error bars show the standard deviation. Panel B shows a western blot analysis using affinity purified antibodies to MLCK. GAPDH was used as a loading control.

**Figure 2:**
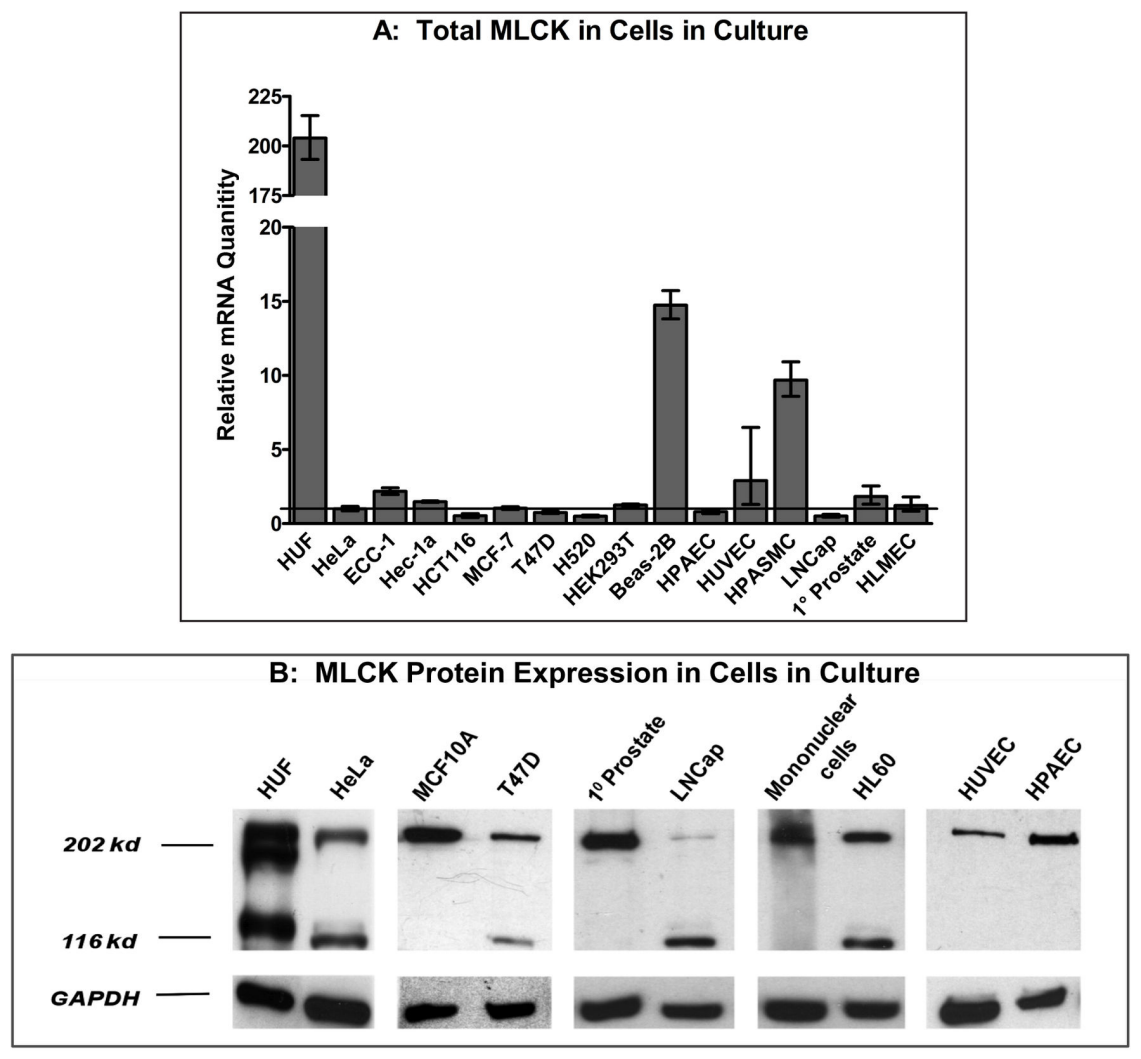
Analysis of MLCK expression in normal and cancer cells in culture. Quantitative PCR to detect total MLCK (A) and western blot (B) analyses of normal and cancer cells in culture. RNA and protein were isolated from various cells and analyzed as described in Figure 1. The data in panel A depict the averages of qPCR analyses performed in triplicate.

We used primers described by Brand-Arpon et al. [23] to analyze the expression of MYLK in human tissues and cells. We obtained tissue from human tumors and the surrounding tissue so that we could compare the expression of MYLK. Quantitative PCR, using primers that target sequences in exons 26 and 27 and recognize all forms of MLCK, revealed MLCK mRNA levels are markedly decreased in bladder, colon, lung, ovary and uterine cancer tissues compared to the normal tissue (Figure 1A). Similarly, qPCR on a broad range of normal human (human uterine fibroblasts, endothelial cells, prostate epithelia and mononuclear cells), non-transformed or transformed cells also revealed lower levels of total MLCK mRNA (Figure 2A) in cancer cells. The data in Figure 2A were normalized to the level of MYLK expression in HeLa cells. Most normal cells (HUF, HPAEC, HUVEC, 1° prostate) and non-tumorigenic Beas-2B cells are above while most cancer cells, with the exception of ECC-1 cells, are below the level of MYLK expression in HeLa cells.

We next determined if protein expression correlated with mRNA levels. MLCK protein expression in normal and cancer tissues and cells was determined by performing western blots using an affinity-purified antibody to MLCK [24]. Normal bladder, colon and uterine tissues expressed both non-muscle and smooth muscle isoforms of MLCK whereas normal lung tissue only expressed smMLCK (Figure 1C). Uniformly, all of the cancer tissues mainly expressed smMLCK and nmMLCK was difficult to visualize in these tissues. Interestingly, the pattern of MLCK protein expression is very similar in normal and lung cancer tissues, although the level of mRNA is decreased in lung cancer tissue (Figure 1). Analysis of tissue culture cells showed that human uterine fibroblasts (HUF cells) express large amounts of both forms of MLCK while human non-muscle cells (endothelial, epithelial and cord blood mononuclear cells) express mostly the larger, nmMLCK (Figure 2B). The expression pattern in cancer cells, however, is complex. Cancer cells (HeLa cervical cancer cells, T47D breast cancer cells, LNCaP prostate cancer cells and HL60 promyelocytic cells), express smMLCK. Interestingly, the increase in smMLCK expression in cancer cells appears to accompany a decrease in the expression of nmMLCK compared to their normal counterparts (eg: compare LNCaP and 1° prostate). Consistent with the PCR data, it is apparent from Figure 2B that the total level of MLCK expression is decreased in cancer cells compared to the control (ie: normal) cells.

**Figure 3:**
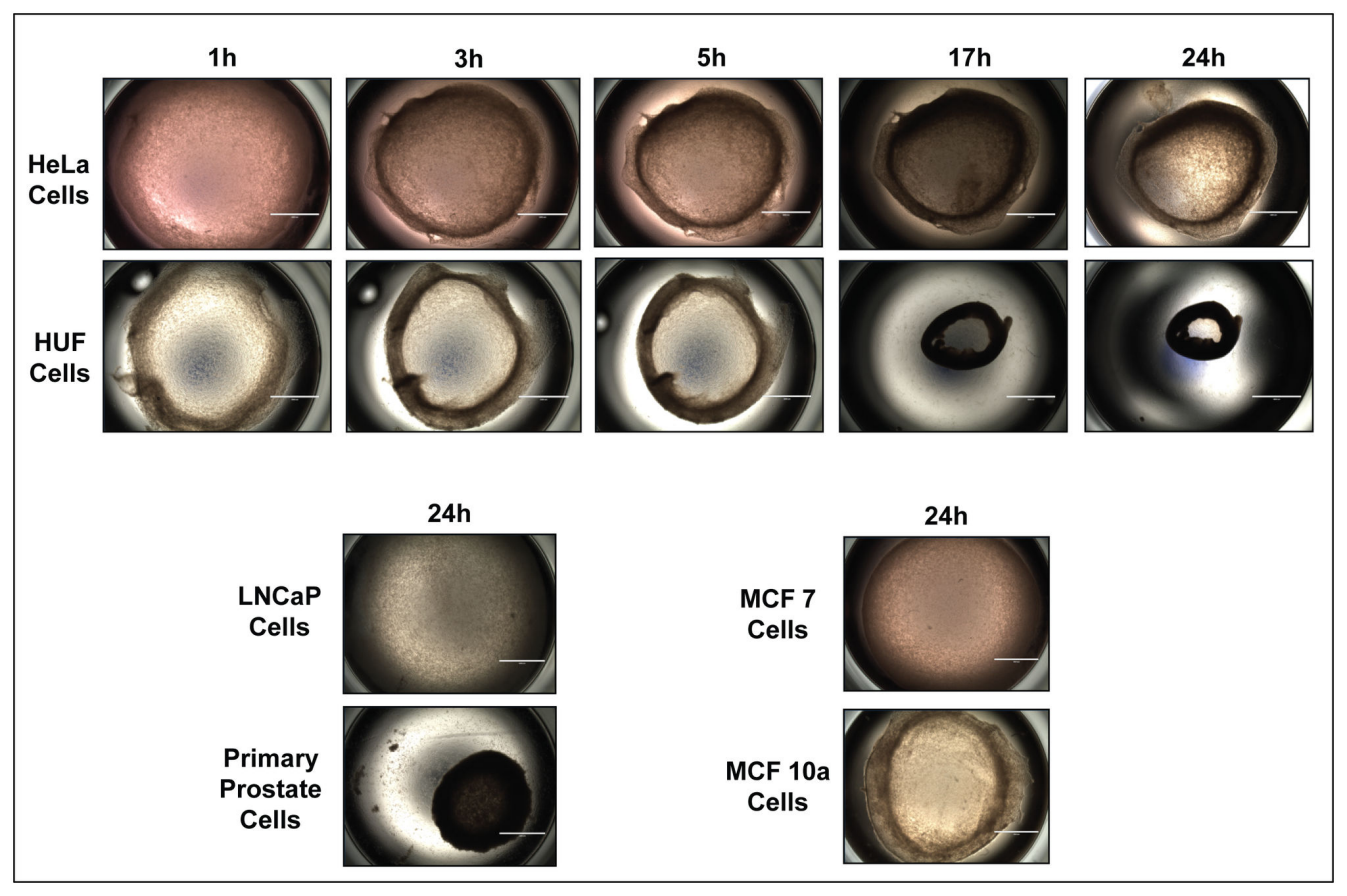
Contraction of 3D collagen gels. HeLa, LNCaP, MCF7, MCF10A, HUF and primary prostate cells were seeded in 24 well dishes in liquid collagen. After the collagen hardened, the gels were released from the sides of the wells and allowed to contract for 24 hrs. The wells were photographed from above at the times shown. Note that the HUF and primary prostate cells contract the gels to a greater extent than HeLa and LNCaP cells and that MCF 10A cells contract more than MCF-7 cells. Scale bar = 2 mm.

We explored the functional consequences of decreased MLCK expression by growing normal and cancer cells in 3D gels and comparing their ability to contract the gels. Liquid collagen containing equal numbers of cells were applied to 24 well dishes and allowed to harden at 37°C. They were then released from the sides of the wells and photographed at defined intervals. Figure 3 shows that HUF and 1° prostate cells contract the gels rapidly. Increasing the collagen concentration to 2 mg/ml decreased the rate and extent of contraction and 3 mg/ml prevented contraction (Figure S1). HeLa cells and MCF10A cells contracted gels to intermediate level whereas MCF7, T47D and LNCap cells barely contracted the gels (Figure 3 and Figure S1).

**Figure 4:**
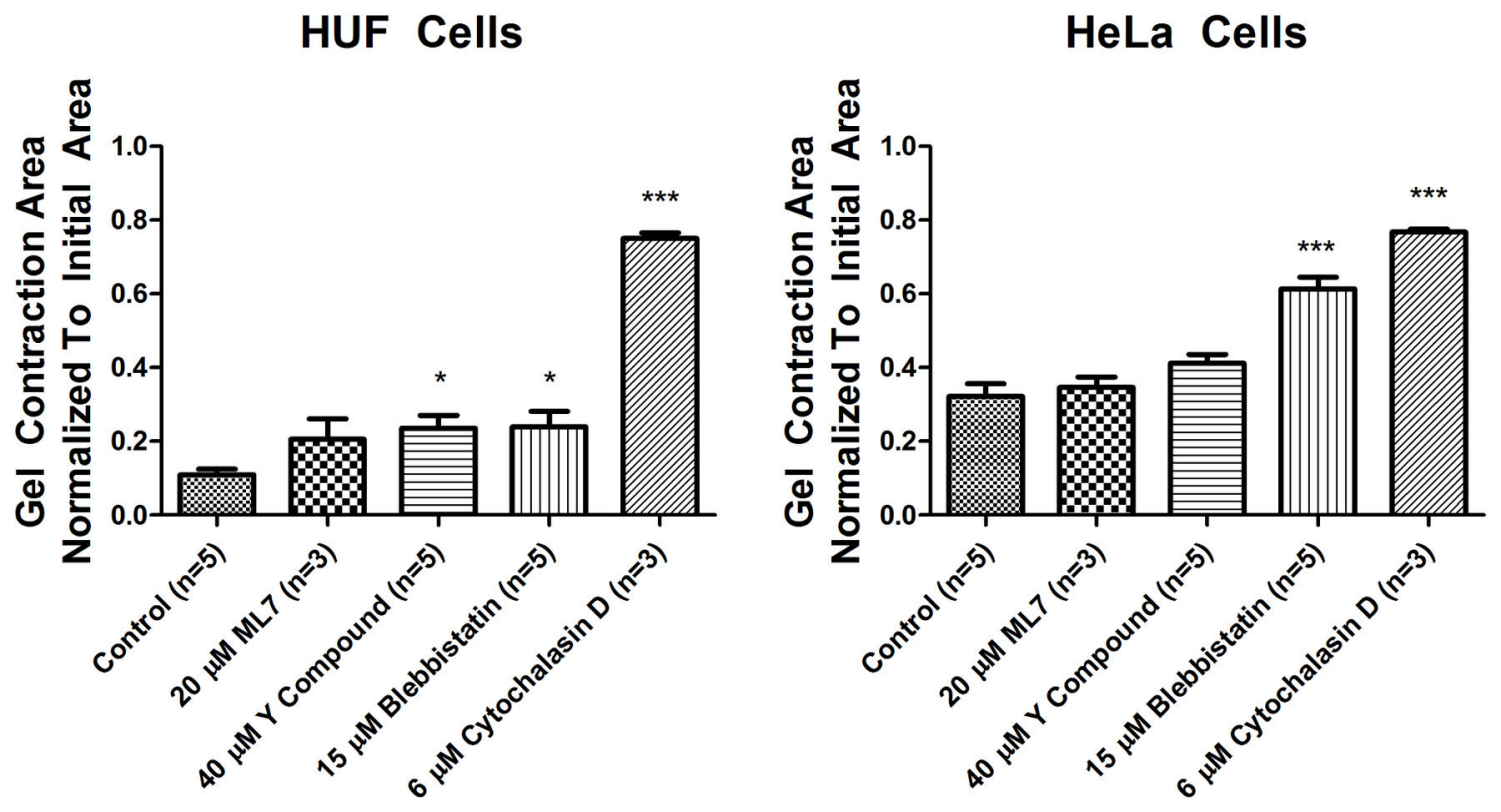
Effect of inhibitors on 3D gel contractions. HUF (left) and HeLa (right) cells were grown in 3D cultures and treated with inhibitors as described. The gels were photographed 24 hrs later and the surface area of the individual gels was quantified. The data represent the mean +SEM. One way ANOVA * = p value < 0.05, *** = p value < 0.001.

We then investigated whether the contractions could be blocked by small molecule inhibitors of the cytoskeleton. We studied HUF and HeLa cells because they contracted the gels. Cells were treated with ML-7, a MLCK inhibitor [32], Y27632, a Rho kinase inhibitor [33], blebbstatin, a myosin II inhibitor [34] and cytochalasin D, which blocks actin polymerization [35]. ML-7 had no effect on contraction of gels containing HUF or HeLa cells (Figure 4). Y27632 and blebbistatin had minimal, albeit statistically-significant, effects on contraction of gels containing HUF cells. Y27632 did not have a statistically significant effect on HeLa cell contraction while blebbistatin had a more pronounced, statistically significant effect on gels made of HeLa cells. Interestingly, cytochalasin D had the most pronounced effect on the gel contractions and almost completely inhibited contractions by both cell types (Figure 4). Washing out the cytochalasin D resulted in a rapid contraction of the gels by both cell types (not shown).

**Figure 5:**
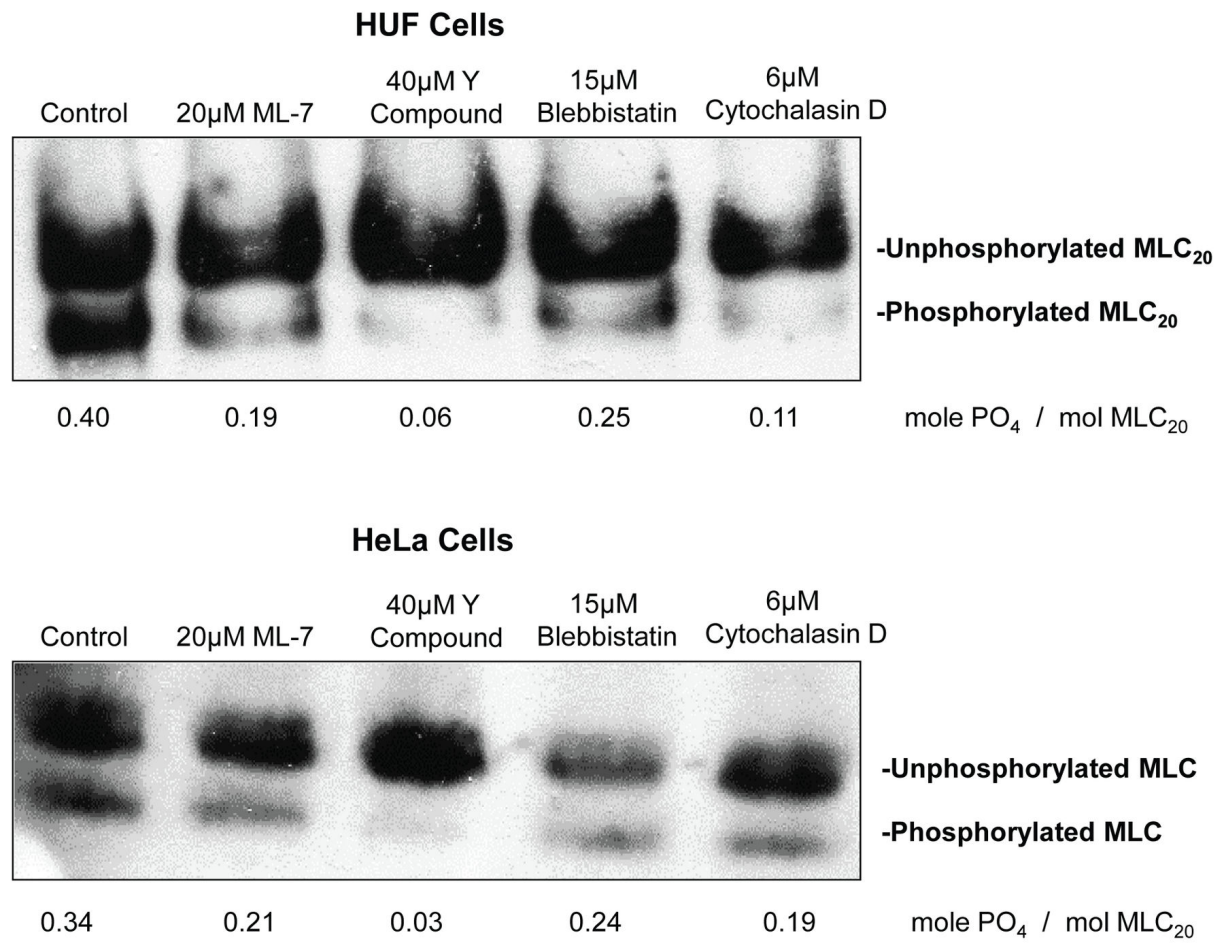
Quantification of MLC phosphorylation of cells treated with inhibitors. The phosphorylated and unphosphorylated MLC were separated by urea-glycerol gel electrophoresis, blotted to nitrocellulose and identified using an antibody the 20 kD MLC.

To establish that ML-7 and Y27632 were in fact inhibiting MLCK and Rho kinase and decreasing MLC phosphorylation, we used glycerol gels to quantify the changes in MLC20 phosphorylation. They showed that MLC20 phosphorylation is decreased in HeLa cell compared to HUF cells and that ML-7 and Y27632 decrease MLC20 phosphorylation (Figure 5). Surprisingly, blebbistatin and cytochalasin D also decrease MLC20 phosphorylation somewhat.

**Figure 6:**
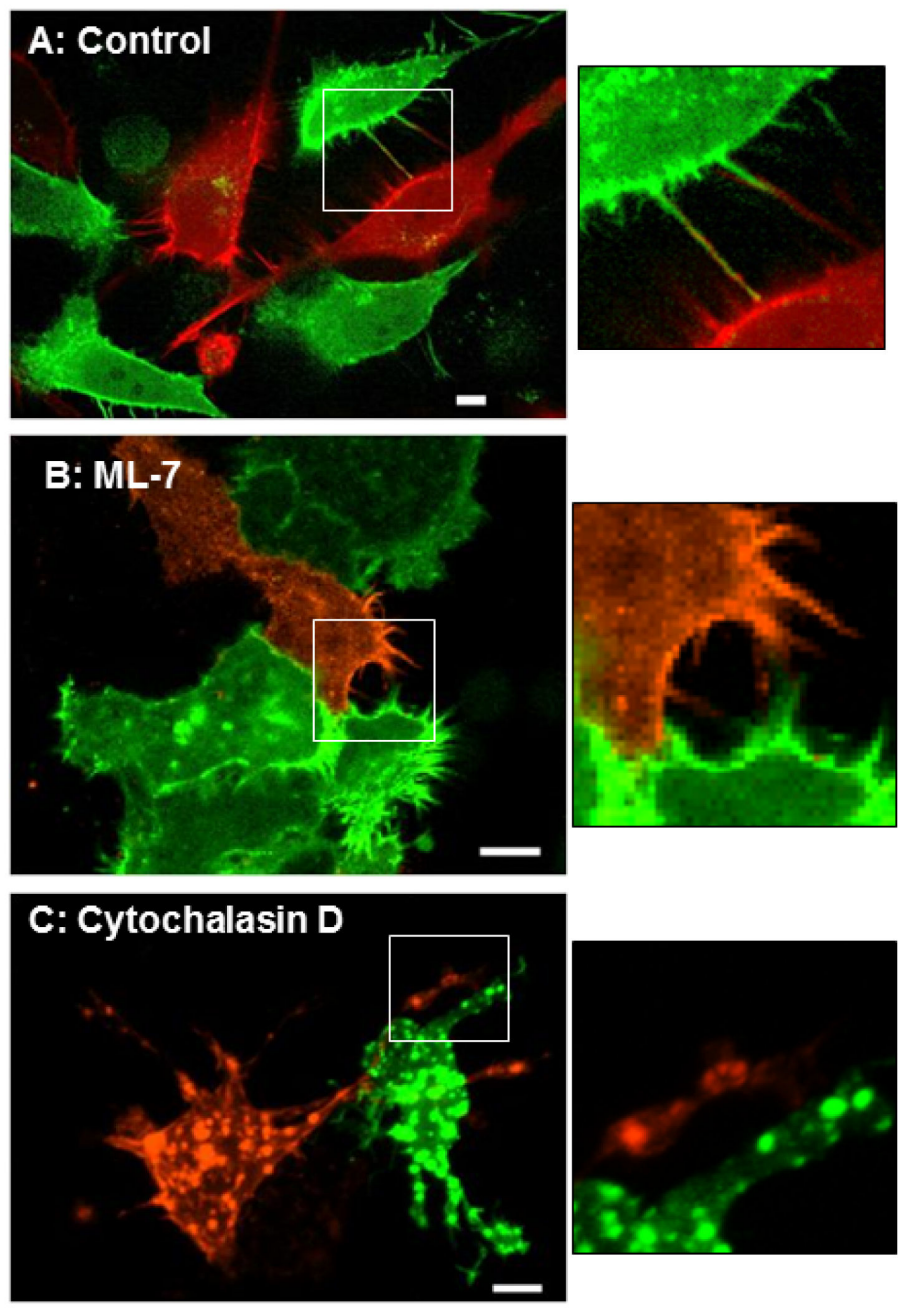
Micrographs of cells grown in collagen gels. HeLa cells were transfected with Lifeact mCherry (red) or Lifeact-GFP and grown in collagen gels. These gels were not released from the walls of the wells to prevent motion artifacts. Panel A shows control (untreated) cells extending filapodia that contact neighboring cells (also see movie in Figure S2). Panels B & C show cells that were treated with 20 μM ML-7 (B) or 6 μM cytochalasin D (C). Cells treated with ML-7 continue to actively extend filopodia. Cells treated with cytochalasin D stop extending filopodia and the actin in these cells appears to collect in large aggregates. The insets are blow ups of the boxed areas. Size bar = 10 μm.

We also used live cell imaging to observe the cells within the gels. These gels were not released from the side of the wells because the motion introduced by the contraction of the gel made it impossible to image the cells. Figure 6 and the movie S1 show that untreated cell are well spread and extend filopodia, which are rich in actin, that reach out and touch each other. Cells treated with ML-7 or Y27632 remained spread and continued to extend filopodia. Cells treated with cytochalasin D remained spread and extended much larger, pseudopodia-like structures rather than filopodia. Importantly, the actin in these cells seemed to coalesce into large aggregates inside the cells.

## Discussion

Tumors can be palpated because they feel harder than the surrounding tissue and many factors, as described in the Introduction, can contribute to the stiffness of a tumor. One of these factors is the contractile state of the cancer cells within a tumor. One of our core assumptions when we began these studies was that cancer cells would up-regulate components of the contractile apparatus. This was based on reports that actomyosin contractility [2] and the expression of rho proteins [4], myosin II [36,37] and MLCK [38] are increased in human tumors relative to normal controls. In addition, when tumor cells metastasize they have to make their way through a forest of collagen fibers and it is reasonable that they would need more activated contractile proteins to push through the extracellular matrix. 

However, a closer examination of the literature suggest otherwise. Experiments using traction force microscopy have found an inverse relationship between force production and metastatic capacity [39]. Mechanical phenotyping using atomic force microscopy [40-42] and other methods [43,44] have consistently shown that cancer cells are softer than normal cells. Moreover, a recent study has suggested that decreased stiffness may improve the survival of cancer cells in the circulation [45]. We have previously shown that over-expressing an active form of MLCK increases MLC phosphorylation and stiffness in fibroblasts [46]. Thus, our observation of a decrease in MLCK expression is very consistent with a decrease in stiffness in transformed cells reported by other laboratories [39-44].

Figure 2 shows that MLCK expression is decreased in tumor cells grown in culture. Nevertheless, tumors are not homogeneous and, in addition to tumor cells, contain immune cells, myofibroblasts and other types of stromal cells that could have increased MLCK levels and thereby contribute to the overall stiffness of the tumor. However, tumor tissues were analyzed in Figure 1 and the data reflect the contributions of all the cell types within each tumor. Although the data clearly demonstrate decreases in MLCK expression compared to the surrounding normal tissue, we cannot eliminate the possibility that increased contractility of non-tumor cells contribute to the stiffness of tumors. One approach for addressing this possibility is to stain tumor tissue with antibodies to MLCK and to quantify the level of staining in cancer cells compared to surrounding normal tissue and non-cancer cells within the mass of the tumor. We are currently establishing the methods to perform such experiments.

The mechanism responsible for down-regulating MLCK expression in cancer cells is not clear. Brand-Arpon et al. [23] have reported the presence of a pseudogene (pMYLK) at chromosome location 3p21 only found in humans, chimps, gorillas and orangutans, but not gibbons or baboons or other species. They also showed that the pMYLK contains a 73 bp deletion and used PCR to differentiate between the expression of MYLK (667 bp) and pMYLK (594 bp). Using the same primers, Han et al. [47] have reported that the expression of pMYLK is increased in cancer cells and the increase in expression of pMYLK is responsible for the decreased expression of MLCK. We used the same primers to analyze the expression of MLCK and pMYLK in human tissues and cells. While we clearly see a decrease in MLCK mRNA and protein expression in cancer cells and tissues, we were not able to consistently detect pMYLK expression in cancer cells or the absence of expression in normal cells. Consequently, the regulation of MLCK expression in transformed cells remains unclear currently.

The observation that the 3D gel contractions are not blocked by ML-7 and Y27632 also warrants comment. An increase in MLC phosphorylation is essential for activating the actin- activated ATPase activity of smooth muscle and non-muscle myosins [9-11]. The partial inhibition by blebbistatin suggests that myosin II cross-bridges play a role in these contractions. However, the inability of ML-7 and Y27632 to block these contractions suggests that MLC phosphorylation is dispensable. In contrast, actin dynamics appear to play a central role in 3D gel contractions. This observation is consistent with the report that the disruption of the actin cytoskeleton blocks all types of collagen gel contractions [48]. Furthermore, the most prominent feature on live cell imaging of cells in the collagen gels (Figure 6) is the presence of highly dynamic actin filopodia. Vonna et al. [49] have studied pathogen capture by macrophages and estimated that filopodia can generate hundreds of pN of force over 10 μm distances. Another study characterized filopodia as “phagocytic tentacles” that retract particles towards the cell body [50]. These authors found the step size of the contractions was 36+13 nm, which excludes myosin II as the possible motor [50]. Although they were not able to implicate any myosin in filopodial retraction, depolymerization of actin filaments with latrunculin A also blocked the filopodial contractions [50]. Taken together, the literature and our data suggest that 3D collagen gel contractions are mediated by actin via filopodia independently of myosin II.

 In summary, our data unequivocally demonstrate that MLCK expression, MLC phosphorylation and 3D gel contraction are lower in cancer cells and tissues than in their normal counterparts. Moreover, we find that decreasing MLC phosphorylation, by inhibiting MLCK or rho kinase, has no effect on 3D gel contractions by normal or transformed cells. In contrast, inhibiting myosin II had a partial effect and depolymerizing actin virtually abolished the contraction. Further, live cell imaging suggested that filopodia play a central role in 3D contractions. These data strongly suggest that tumor rigidity, the underlying basis of self-detection of tumors, is independent of myosin II phosphorylation. Our observations are consistent with recent studies that have shown that cancer cells are less stiff than non-transformed cells [40-44]. Lastly, we have previously shown that inhibiting MLCK induces apoptosis in vitro and potentiates the effects of anticancer drugs to induce apoptosis and inhibit tumor growth in vivo [17]. Our current demonstration that MLCK expression is decreased in tumor cells suggests a targeting window for specifically inducing apoptosis in cancer cells and provokes further investigation of MLCK as a potential therapeutic target.

## Supporting Information

Figure S1 **Summary of collagen gel contraction assays.** The gels were incubated for 24 hours, photographed from above and surface area calculated as described in methods. Panel A shows that HUF and primary prostate cells contract the gels more than HeLa and LNCap cells. MCF10A breast cancer cells also contract the gels more than the more aggressive MCF7 and T47D cancer cells. Panel B shows that increasing the collagen concentration inhibits contraction dose dependently. N = 2, +/- SD, * and *** equal p values of <0.05 and <0.001, respectively.
(TIF)

Movie S1 **Movie of control HeLa cells in a collagen gel.**
(MOV)
