# Supplementary figures and images for: Tumor Stiffness Is Unrelated to Myosin Light Chain Phosphorylation in Cancer Cells
Source: PLoS One. 2013 Nov 4;8(11):e79776. doi: 10.1371/journal.pone.0079776 (PMC3817105; doi:10.1371/journal.pone.0079776)

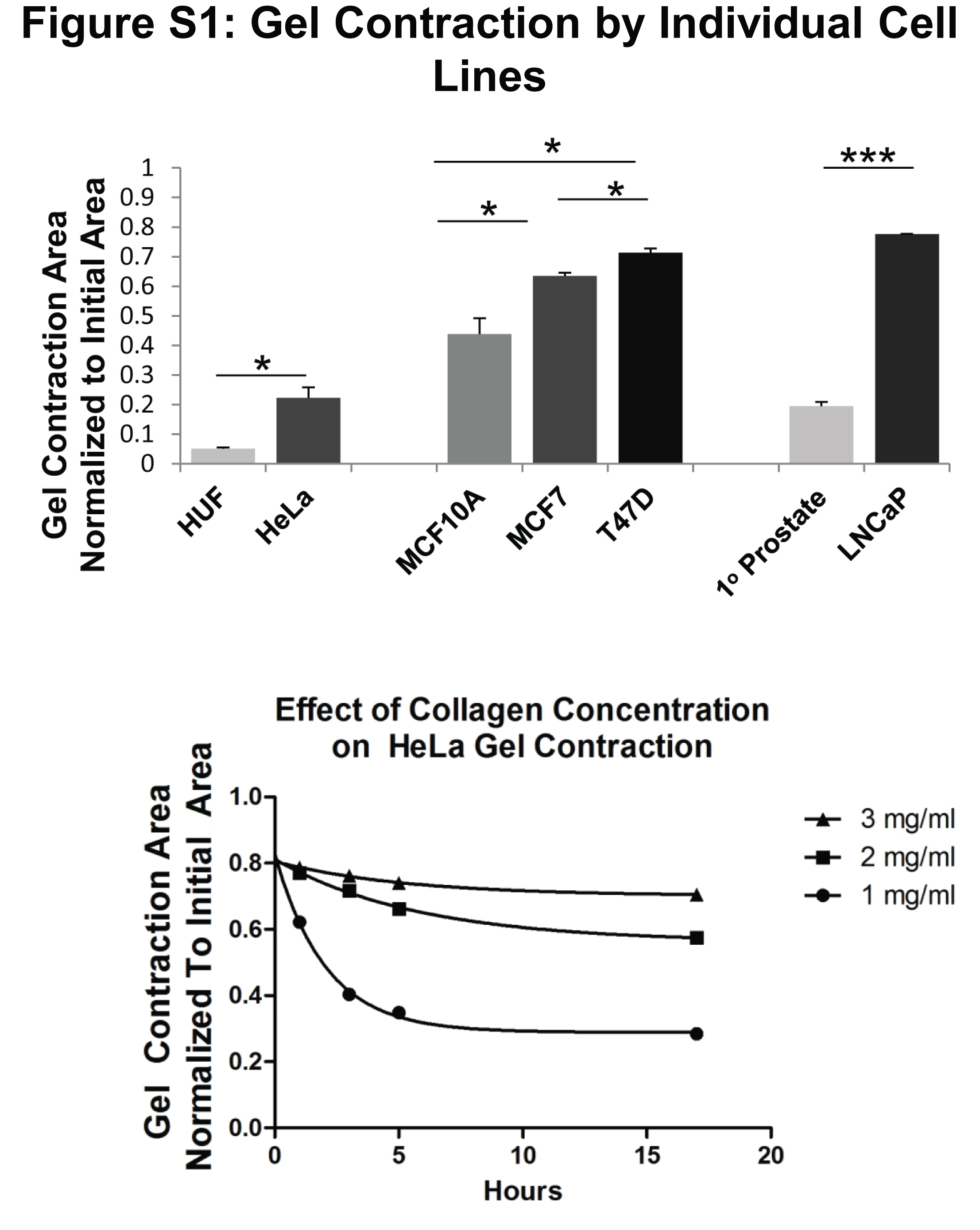

Supplement: Figure S1 — Summary of collagen gel contraction assays. The gels were incubated for 24 hours, photographed from above and surface area calculated as described in methods. Panel A shows that HUF and primary prostate cells contract the gels more than HeLa and LNCap cells. MCF10A breast cancer cells also contract the gels more than the more aggressive MCF7 and T47D cancer cells. Panel B shows that increasing the collagen concentration inhibits contraction dose dependently. N = 2, +/- SD, * and *** equal p values of <0.05 and <0.001, respectively. (TIF) [file pone.0079776.s001.tif]
